# Robust quantification of ICG fluorescence perfusion in neonatal bowel surgery via deep point tracking

**DOI:** 10.1007/s11548-026-03733-w

**Published:** 2026-06-16

**Authors:** Zhehua Mao, Antonio Composto, Jonathan Neville, Cecilia Cirelli, Danail Stoyanov, Stefano Giuliani, Sophia Bano

**Affiliations:** 1 Department of Computer Science, University College London, London, UK; 2 UCL Hawkes Institute, University College London, London, UK; 3 Great Ormond Street Hospital for Children NHS Trust, London, UK

**Keywords:** ICG fluorescence imaging, Point tracking, Bowel perfusion, Quantitative analysis, Surgical guidance

## Abstract

****Purpose**:**

Indocyanine green (ICG) fluorescence imaging is increasingly used for intraoperative bowel perfusion assessment in neonatal surgery. However, existing commercial solutions are limited to track few manually selected regions of interest (ROIs) and struggle with tissue motion and occlusion. We present a novel ICG quantification framework leveraging deep learning-based point tracking for robust perfusion analysis.

****Methods**:**

Our system employs CoTracker3, a state-of-the-art transformer-based point tracking model, to enable tracking of both user-specified ROIs and dense sampling across the entire visible tissue region. The framework computes comprehensive perfusion metrics including $F_{\max }$, $T_{\max }$, ingress gradient, $T_{1/2\max }$, and washout rate for each tracked region. For dense tissue analysis, dynamic perfusion heatmaps are generated through spatial interpolation with concave hull boundary reconstruction.

****Results**:**

Preliminary validation on neonatal bowel perfusion videos demonstrates robust tracking performance under tissue motion and partial occlusion. The system successfully extracts perfusion metrics comparable to the commercial PerfusionTech system while enabling dense spatial perfusion mapping not available in existing solutions.

****Conclusion**:**

The proposed CoTracker3-based framework provides a feasible approach for quantitative ICG fluorescence analysis with improved tracking robustness and comprehensive spatial perfusion visualization, warranting further clinical validation studies.

## Introduction

**Fig. 1:**
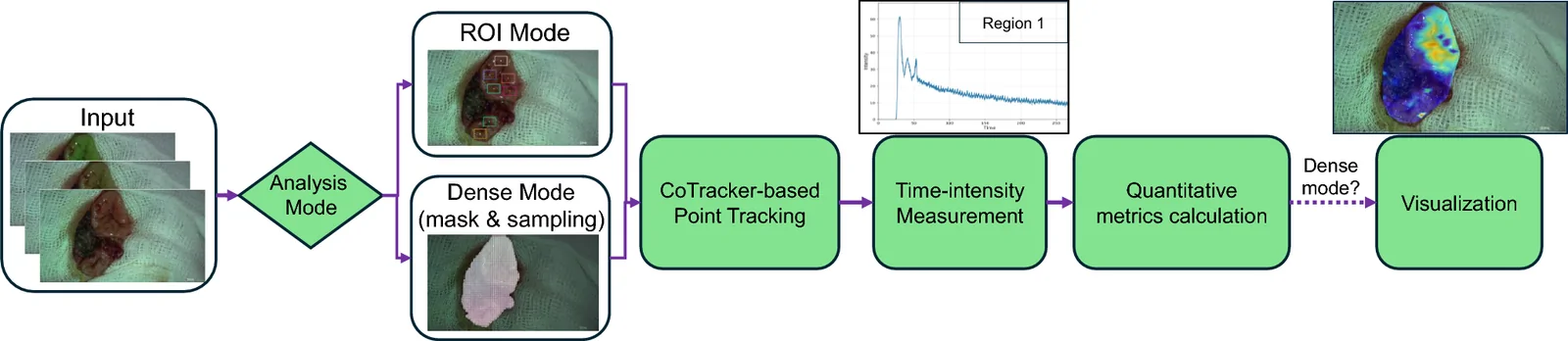
System overview. The pipeline supports both ROI-based quantification and dense tissue mapping

**Fig. 2:**
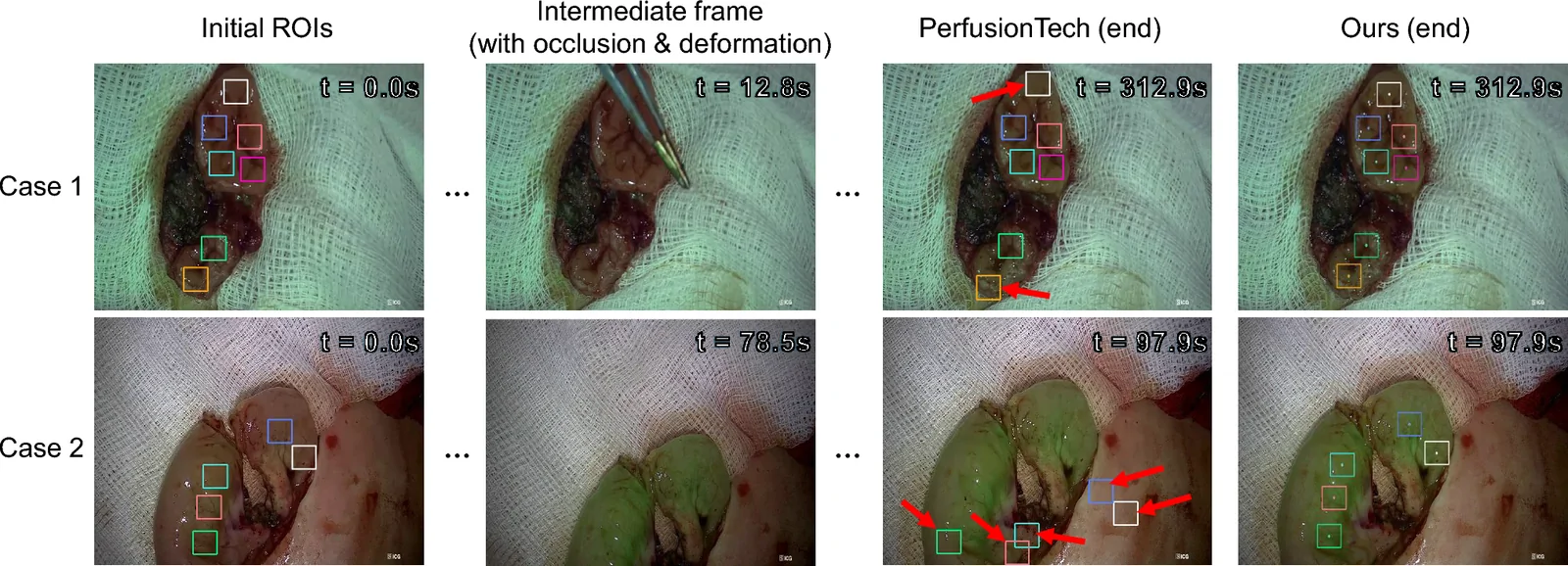
Two representative tracking examples. The first column shows the initial ROI placement at the start of ICG perfusion measurement (t=0.0 s), where our ROIs are placed at the same positions and with the same sizes as those used by PerfusionTech. The second column shows an intermediate frame during the measurement, illustrating surgical instrument occlusion and tissue deformation/movement. The third and fourth columns show the final tracking results from PerfusionTech and our method, respectively. Relative timestamps are provided in the top-right corner of each frame to indicate the progression from the start of the sequence. Due to occlusion and deformation, PerfusionTech fails to maintain accurate tracking for several ROIs (indicated by red arrows), whereas the proposed method preserves accurate correspondence, with tracked regions closely matching their initial placement

Indocyanine green (ICG) fluorescence angiography is increasingly used for intraoperative assessment of bowel perfusion, particularly in neonatal and pediatric surgery where defining viable resection margins is critical [1, 2]. In this technique, ICG (a near-infrared fluorescent dye) is injected intravenously during surgery, and a near-infrared camera captures the dye’s transit through the tissue vasculature in real time, producing a dynamic fluorescence video of blood perfusion directly at the operative site [1]. This allows surgeons to evaluate tissue viability and identify ischemic or poorly perfused zones *in situ*, which is critical for determining safe anastomotic or resection margins (e.g., in necrotizing enterocolitis).  However, visual interpretation remains subjective and sensitive to camera motion, tissue deformation, and transient occlusions. Quantitative metrics derived from the fluorescence time-intensity curves, such as peak intensity ($F_{\max }$), ingress slope, and washout rate, provide objective, reproducible parameters that can capture subtle differences in perfusion dynamics beyond what qualitative visual assessment can offer, thereby supporting standardized clinical decision-making [1]. Commercial tools (e.g., PerfusionTech) offer quantitative analysis by extracting time–intensity curves from a small number of manually placed regions of interest (ROIs) [1], but they are typically limited to a few ROIs and their motion compensation can fail under non-rigid deformation or occlusion, compromising measurement reliability and limiting spatial assessment.

**Table 1:** Comparison of perfusion metrics between proposed method and PerfusionTech (N=21 ROIs from 3 patients). LoA: limits of agreement (Bland–Altman [6])

Metric	Mean ± SD		*r*	Bias	LoA
	Proposed	PerfusionTech			
$F_{\max }$ (AU)	49.88±14.35	48.20±14.79	0.86	1.68	[−13.63, 16.99]
$T_{\max }$ (s)	51.66±16.33	51.69±16.31	**1**.**00**	−0.02	[−0.75, 0.70]
$T_{\text {rise}}$ (s)	46.74±15.80	47.64±16.29	**1**.**00**	−0.90	[−2.07, 0.27]
Gradient (AU/s)	10.85±4.23	13.77±6.87	0.97	−2.92	[−8.91, 3.08]
$T_{1/2\max }$ (s)	48.75±16.05	49.02±16.15	**1**.**00**	−0.26	[−0.97, 0.44]
Washout Rate (AU/s)	0.82±0.29	1.09±0.38	0.98	−0.27	[−0.49, −0.05]

Recent advances in deep learning-based point tracking have substantially improved the robustness of long-term tracking under challenging conditions such as large motions and occlusions [3, 4]. In particular, CoTracker3 [4] is a state-of-the-art method that employs a transformer-based architecture to jointly track multiple points while enforcing temporal consistency. It enables reliable sparse and dense point tracking over long sequences, explicitly accommodating point disappearance and reappearance. We leverage these strengths to achieve robust tissue tracking in intraoperative fluorescence videos, thereby supporting reliable ICG fluorescence quantification and spatially resolved perfusion mapping. Our contributions include:

1. A dual-mode ICG quantification framework that uses a state-of-the-art point tracker to maintain correspondence of ROIs/points under non-rigid tissue motion and occlusions, enabling reproducible extraction of perfusion parameters.
2. Dense tissue-level perfusion mapping, producing spatial heatmaps of clinically used perfusion metrics beyond a small set of discrete ROIs.
3. A preliminary clinical evaluation on neonatal bowel perfusion videos, including a direct comparison against a commercial solution, demonstrating improved tracking robustness and strong agreement in extracted metrics.

## Methodology

**Overview.** Given an ICG fluorescence video, the pipeline (Fig. 1) performs: (i) point/ROI initialization, (ii) long-term point tracking, (iii) fluorescence signal extraction and feature computation, and (iv) visualization.

**Fig. 3:**
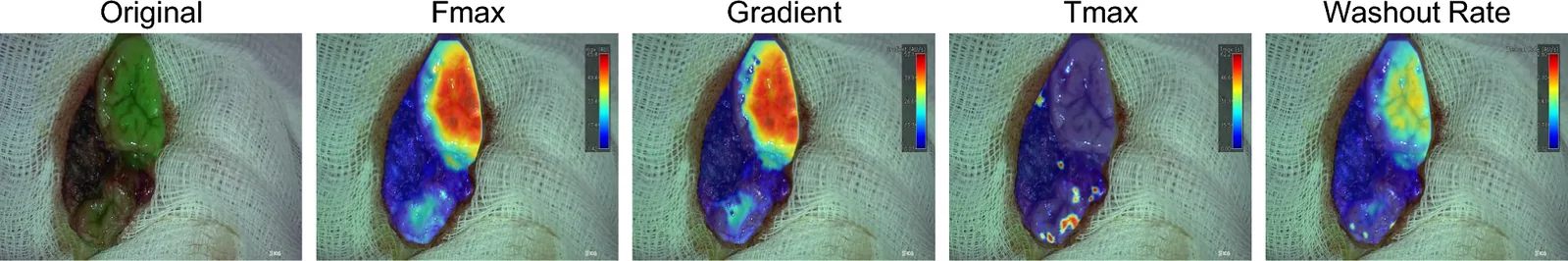
One example of qualitative dense mapping results, i.e., heatmaps of $F_{\max }$, *Gradient*, $T_{\max }$, and Washout Rate

**Dual-mode sampling.** *(1) ROI mode:* To mirror clinical workflows and enable direct comparison with commercial tools, users specify rectangular ROIs by center $(x_c,y_c)$ and size (*w*, *h*). We track ROI centers over time and extract fluorescence within each ROI. *(2) Dense mapping mode:* To obtain spatially resolved perfusion information, we segment the visible bowel region in the first frame using simple HSV thresholding (optimized for bowel tissue against gauze/background). A regular grid of points is then sampled inside the mask. Grid spacing is controlled by patch size *p* and overlap ratio

$$\rho : s = p(1-\rho ).$$

**CoTracker3-based tracking.**   All sampled points are tracked using CoTracker3 [4], which jointly tracks all points with a transformer-based model and predicts point visibility. This is particularly suited to fluorescence angiography videos where instruments and tissue manipulation often cause occlusion and large non-rigid motion. The tracked trajectories provide motion compensation for subsequent fluorescence measurement.

**Time–intensity extraction and normalization.** For each tracked point *i*, we measure the mean intensity within its associated ROI at each time *t*, yielding $I^{(i)}(t)$. To remove baseline fluorescence, we compute the mean of the first $N_b$ frames:

$$\begin{aligned} I_{\text {baseline}}^{(i)}=\frac{1}{N_b}\sum _{t=1}^{N_b} I^{(i)}(t).\end{aligned}$$

The baseline-corrected intensity is then calculated by:

$$\begin{aligned} I_{\text {corr}}^{(i)}(t)=I^{(i)}(t)-I_{\text {baseline}}^{(i)}.\end{aligned}$$

To reduce noise while preserving curve shape, we apply Savitzky–Golay smoothing [5].

**Perfusion metrics and Dense visualization.** Following commonly reported clinical metrics [1], we compute $F_{\max }$, $T_{\max }$, ingress gradient, $T_{1/2\max }$, and washout rate from the smoothed time–intensity curves. Specifically,

- $F_{\max }$ (peak fluorescence intensity): reflects the maximum perfusion volume reaching the tissue.
- $T_{\max }$ (time to peak): characterizes perfusion speed, prolonged values may indicate impaired arterial supply.
- Ingress gradient (average rate of fluorescence rise): captures microvascular inflow dynamics, low slopes suggest microcirculation disorders.
- $T_{1/2\max }$ (time to half-maximum): describes perfusion onset characteristics.
- Washout rate (fluorescence decline after peak): reflects venous return, absence of washout may indicate venous obstruction.

In dense mapping mode, per-point metrics are interpolated over the tissue region to generate spatial heatmaps, enabling inspection of perfusion heterogeneity and potential border zones.

## Preliminary Results and Discussion

**Dataset.** We evaluated on ICG bowel perfusion videos from six neonatal patients acquired using a Storz Rubina Lens (1920×1080, 25 fps).

**Tracking robustness.** We reproduced the ROI placement from PerfusionTech by importing its exported ROI position/size and initialization timestamp and then assessed whether the ROI remained aligned with the intended tissue region until the end of the measurement interval. Across six cases, PerfusionTech exhibited frequent drift or loss under deformation, camera motion, and intermittent occlusion (Fig. 2). In contrast, CoTracker3-based tracking maintained stable correspondence throughout these challenging segments, supporting its suitability for reliable quantification.

**Agreement with PerfusionTech metrics.** For quantitative comparison, we excluded three cases where PerfusionTech failed to maintain ROI tracking and analyzed three remaining cases. We evaluated seven matched ROIs per case (21 ROI pairs). Table 1 reports Pearson correlation (*r*), mean bias, and Bland–Altman 95% limits of agreement (LoA). We observe near-perfect agreement for temporal metrics ($T_{\max }$, $T_{\text {rise}}$, $T_{1/2\max }$) and strong concordance for intensity- and slope-related measures, indicating that the proposed motion-compensated pipeline preserves clinically relevant fluorescence dynamics while improving robustness in difficult sequences.

**Dense perfusion mapping.** Beyond a small set of ROIs, dense sampling provides spatial maps of perfusion metrics over the visible bowel surface (Fig. 3). This reduces dependence on manual ROI placement and enables interactive inspection of heterogeneous perfusion patterns that may be missed when analyzing only a few discrete regions.

## Conclusion

We introduced a CoTracker3-enabled framework for quantitative ICG fluorescence analysis that supports both ROI-based measurements and dense tissue-level perfusion mapping. Preliminary results on neonatal bowel videos show improved robustness to deformation and occlusion compared with a commercial tool, while maintaining strong agreement in extracted perfusion metrics. Future work will focus on larger-scale validation and optimization toward real-time deployment.
